# Effect of Vectashield-induced fluorescence quenching on conventional and super-resolution microscopy

**DOI:** 10.1038/s41598-020-63418-5

**Published:** 2020-04-15

**Authors:** Aleksandra Arsić, Nevena Stajković, Rainer Spiegel, Ivana Nikić-Spiegel

**Affiliations:** 1 Werner Reichardt Centre for Integrative Neuroscience, University of Tübingen, Otfried-Müller-Straße 25, 72076 Tübingen, Germany; 2 BG Hospital Tübingen,University of Tübingen, Schnarrenbergstraße 95, 72076 Tübingen, Germany

**Keywords:** Fluorescence imaging, Fluorescence imaging, Confocal microscopy, Confocal microscopy, Super-resolution microscopy

## Abstract

Finding the right combination of a fluorescent dye and a mounting medium is crucial for optimal microscopy of fixed samples. It was recently shown that Vectashield, one of the most commonly used mounting media for conventional microscopy, can also be applied to super-resolution direct stochastic optical reconstruction microscopy (dSTORM). dSTORM utilizes conventional dyes and starts with samples in a fluorescent “ON” state. This helps in identifying structures of interest. Subsequently, labelled samples are induced into blinking, which is necessary for determining the position of single molecules and reconstruction of super-resolution images. This is only possible with certain fluorescent dyes and imaging buffers. One of the most widely used dyes for dSTORM, Alexa Fluor 647 (AF647), blinks in Vectashield. However, after preparing immunocytochemical samples in Vectashield, we noticed that the fluorescence intensity of AF647 is quenched. This is particularly evident for dimmer immunostainings, such as stainings of some components of neuronal cytoskeleton and axonal initial segment. Because structures of interest cannot be identified in quenched samples, loss of fluorescence intensity hinders imaging of AF647 in Vectashield. This has consequences for both conventional and dSTORM imaging. To overcome this, we provide: 1) a quantitative analysis of AF647 intensity in different imaging media, 2) a quantitative analysis of the suitability of Vectashield for dSTORM imaging of high and low-abundance AF647-labelled targets. Furthermore, for the first time, we quantitatively analyse the performance of Alexa Fluor Plus 647, a new variant of AF647-conjugated antibody, in dSTORM imaging.

## Introduction

Super-resolution microscopy (SRM) provides unique insights into the subcellular organisation of cells and tissues. Several techniques have been developed to make nanoscale imaging possible^1–4^. Single-molecule localisation techniques, such as photoactivated localisation microscopy (PALM)^5^, fluorescence photoactivation localisation microscopy (FPALM)^6^, stochastic optical reconstruction microscopy (STORM)^7^ and direct stochastic optical reconstruction microscopy (dSTORM)^8^ rely on temporal separation of fluorescence emission. Temporal separation is achieved by blinking, which separates individual fluorescent molecules not only in time, but also in space. The resulting spatiotemporal separation of fluorescence emission enables precise determination of the location of fluorescent molecules, which is necessary to reconstruct SRM images^7,9^. Whereas PALM and FPALM utilize genetically engineered fluorescent proteins, dSTORM relies on synthetic fluorophores (dyes). To achieve blinking in dSTORM, dye molecules are switched to a dark “OFF” state from which they stochastically and spontaneously recover to a fluorescent “ON” state, multiple times before bleaching. Blinking can be induced in certain dyes by using strong lasers and particular imaging buffers, such as thiol-containing reducing agents (e.g. β-mercaptoethanol) with or without enzymatic oxygen scavenger systems (e.g. GLOX buffer containing glucose oxidase and catalase)^8,10^.

Blinking is essential for dSTORM/FPALM, and having the dye molecules in the fluorescent ON state before the induction of blinking is of high importance for optimal imaging. Being able to detect the fluorescent signal is necessary for the identification of positively labelled cells and structures of interest. This is relevant for transfection-based FPALM experiments in which, depending on the efficiency, only a certain number of cells will be transfected and thus can be imaged under a microscope. This is equally relevant for immunocytochemistry/immunohistochemistry-based dSTORM, in which specific regions of cells or tissues need to be identified. In addition, having the dye molecules in the fluorescent ON state is necessary for the assessment of the quality of labelling. Furthermore, it is normal practice in dSTORM and other SRM techniques to take a reference image with conventional diffraction-limited microscopy prior to switching to the SRM mode. For this, fluorescent molecules need to be in the ON state. Last but not least, having molecules in the ON state is necessary for setting a correct focal point for 3D dSTORM imaging^11^.

It was recently reported that Vectashield, a commercial mounting medium for confocal microscopy, can also be used as an imaging buffer for structured illumination microscopy (SIM)^12^, dSTORM^13^ or their combination^14^. In comparison with consumer-made buffers, Vectashield is a convenient and affordable medium for dSTORM imaging. The fact that it can be used as purchased is particularly beneficial for laborious techniques such as dSTORM. Using the same commercially available imaging medium offers more reproducibility and allows for better comparability of results within and between laboratories. In addition, Vectashield has a high refractive index (1.45) and can be used for imaging with objectives with high numerical apertures. Another advantage is that Vectashield is stable and can be used for long imaging sessions. Owing to the depletion of its oxygen scavenging system, GLOX buffer can only be used for a couple of hours, whereas samples can stay in Vectashield for several days^13^.

In the first report on the suitability of Vectashield for dSTORM imaging^13^, the authors imaged microtubules and tested several fluorescent dyes, including Alexa Fluor 647 (AF647), Alexa Fluor 700 (AF700), Alexa Fluor 555 (AF555) and Cy3. The best blinking was achieved with AF647. Combining Vectashield and AF647 for dSTORM is of the highest practical importance because AF647 is the most widely used dye for dSTORM. However, this is in contrast to a single previous report on the incompatibility of AF647 and Vectashield^15^. Although Olivier and colleagues point out that Vectashield is not suitable for imaging with every dye, they do not mention potential issues with imaging AF647 in Vectashield, nor are any mentioned in other reports^14,16–18^.

The reason for this could be that until now, only a few cellular targets have been imaged in Vectashield with dSTORM; these were mainly cytoskeletal elements, such as actin and microtubules. These high-abundance cellular structures are relatively easy to image and usually serve as proof-of-principle target proteins for comparing different SRM techniques or imaging conditions. In our hands, imaging of these proteins in Vectashield was also straightforward, but this was not the case when using Vectashield for the imaging of dimmer immunostainings (or stainings of less abundant proteins). Specifically, we could not identify AF647-labelled components of neuronal cytoskeleton and axonal initial segment in Vectashield. This was intriguing because the same samples showed positive immunofluorescence labelling in phosphate-buffered saline (PBS), suggesting a quenching of the AF647 ON state in Vectashield. To understand this, we performed a quantitative analysis of the fluorescence intensity of AF647 and the new variant Alexa Fluor Plus 647 (AF(+)647) in Vectashield. Finally, based on our quantitative analysis, we could optimize imaging conditions in Vectashield to obtain 3D dSTORM images of less-abundant targets, such as neurofilaments in a neuronal cell line, ßII spectrin and components of axonal initial segment (ankyrin G, voltage-gated sodium channels) in primary mouse neurons. Furthermore, we compared the suitability of Vectashield and diluted Vectashield (25% in Tris-glycerol) for dSTORM imaging of AF647- and AF(+)647-labelled high- and low-abundance targets.

## Results

**Figure 1:**
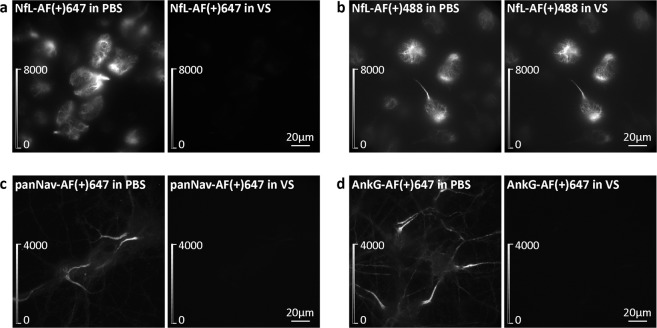
Effect of Vectashield (VS) on AF(+)647 and AF(+)488 fluorescence intensity. Widefield images of (**a**) AF(+)647-labelled neurofilament light chain (NfL) in ND7/23 cells, (**b**) AF(+)488-labelled NfL in ND7/23 cells, (**c**) AF(+)647-labelled voltage-gated sodium channels (panNav) in mouse cortical neurons (MCN), (**d**) AF(+)647-labelled ankyrin G (AnkG) in MCN. In panels (**a**–**d**), the same field of view is shown for PBS and VS conditions. Brightness and contrast were linearly adjusted to show the same display range in both PBS and VS conditions, as indicated by the look-up table (LUT) intensity scale bars.

The aim of our study was to image neuronal proteins with dSTORM. Considering long imaging sessions, we wanted to find a buffer which is more stable than the conventional GLOX buffer. Vectashield seemed like a good alternative^13^. At the same time, we wanted to test the suitability of the new AF(+)647-conjugated secondary antibody for dSTORM imaging. To achieve this, we first established dSTORM imaging of microtubules in Vectashield (Supplementary Fig. 1), as described in the literature^13^. Based on these conditions, we attempted to image AF(+)647-labelled neurofilament light chain (NfL) in neuronal ND7/23 cells and components of axonal initial segments in primary mouse cortical neurons. To our surprise, this was not possible at first, due to the substantial loss of AF(+)647 fluorescence in Vectashield compared to phosphate-buffered saline (PBS) (Fig. 1a,c,d). A control experiment showed that the fluorescence emission intensities of both AF647 and AF(+)647 decreased after the PBS was exchanged for Vectashield (Supplementary Fig. 2), such that the NfL and axonal initial segments labelled with these dyes looked completely dark. Since the dyes were quenched in the fluorescent ON state, it was difficult to distinguish labelled cells from the background. To exclude the possibility of antibody washout that is associated with changing the imaging medium, we performed a control experiment in which PBS was exchanged for fresh PBS. This experiment showed no decrease of fluorescence intensity (Supplementary Fig. 3). As an additional control, we showed that Vectashield does not quench Alexa Fluor Plus 488 (AF(+)488; Fig. 1b) or other orange and red dyes, such as AF555 and Alexa Fluor 633 (AF633; Supplementary Fig. 4).

**Figure 2:**
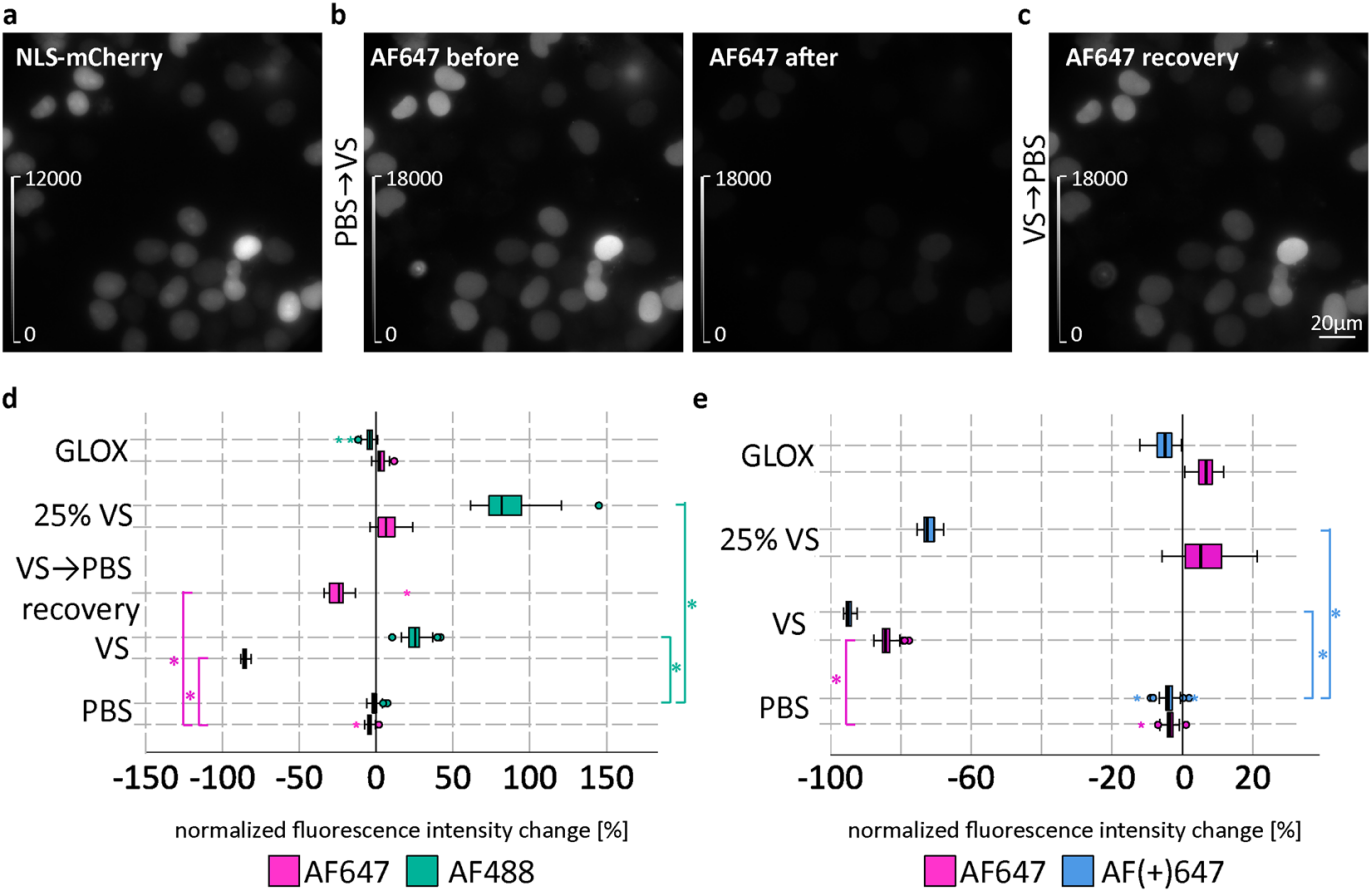
Quantification of Vectashield (VS)-induced fluorescence intensity changes. (**a**) Example widefield image of ND7/23 cells expressing NLS (nuclear localisation sequence)-mCherry. Cells were immunolabelled with anti-tRFP primary antibody, followed by AF647-conjugated secondary antibody. (**b**) Same field of view in the AF647 channel before and after changing the medium from PBS to VS. (**c**) Same field of view in the 647 channel in PBS 2.5 h after removal of VS. Brightness and contrast were linearly adjusted to show the same display range in different conditions (as indicated by look-up table (LUT) intensity scale bars). (**d**,**e**) Box plots show the comparison of normalized intensity changes in different imaging media for AF647 versus AF488 (**d**) and AF647 versus AF(+)647 (**e**) dyes. Data were pooled from three independent experiments (*n* = 20 images per medium per dye per experiment). Circles indicate outliers. * indicates significance with *p* < 0.05, using separate analyses of variance (ANOVAs) with post-hoc Bonferroni comparisons. Note that the normalized fluorescence intensity changes were used for clarity in the figure. The actual dependent variables of the ANOVAs were delta intensities. Detailed explanations on how the dependent variables were calculated can be found in the Supplementary Information.

To quantify Vectashield-induced fluorescence intensity changes, ND7/23 cells were transfected with a plasmid encoding a nuclear localisation sequence (NLS)-mCherry fusion protein (Fig. 2a). We chose to use NLS-mCherry for intensity measurements for multiple reasons. First, it is uniformly distributed within the nucleus, and is readily labelled with anti-mCherry primary antibody, followed by either AF647-, AF(+)647- or AF488-conjugated secondary antibody. Furthermore, mCherry gives a bright fluorescent signal in the 561 nm (mCherry) channel that is not affected by any of the imaging media tested. This can be used for reliable identification of labelled regions of interest and intensity measurements of AF647 and AF488 before and after changing the imaging medium (Fig. 2b and Supplementary Fig. 5). Quantitative analysis of fluorescence intensity changes shows that in Vectashield, there is an intensity drop to about 15% of the initial value for AF647 and to about 5% for AF(+)647, and no loss of intensity for AF488 (Fig. 2d,e). We could also show that the quenched AF647 signal recovers after a washing step **(**Fig. 2c,d).

**Figure 3:**
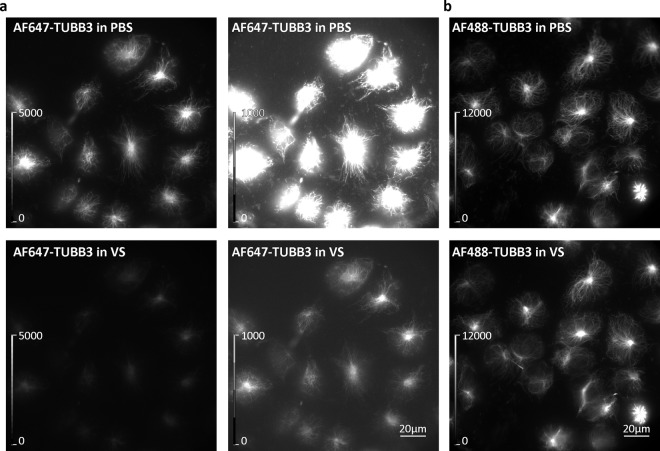
Effect of Vectashield (VS) on the fluorescence of AF647- and AF488-labelled microtubules. (**a**) Widefield images of ND7/23 cells labelled with AF647-conjugated anti-tubulin β3 (AF647-TUBB3) antibody in PBS (upper panels) and in VS (lower panels). To ensure the quenched AF647 signal in VS is visible, the same images are shown on the right with different linearly adjusted brightness and contrast levels (as indicated by look-up table (LUT) intensity scale bars). (**b**) Widefield images of ND7/23 cells labelled with AF488-conjugated anti-tubulin β3 (AF488-TUBB3) antibody in PBS (upper panel) and in VS (lower panel). Brightness and contrast were linearly adjusted to show the same display range in both PBS and VS conditions (as indicated by LUT intensity scale bars).

**Figure 4:**
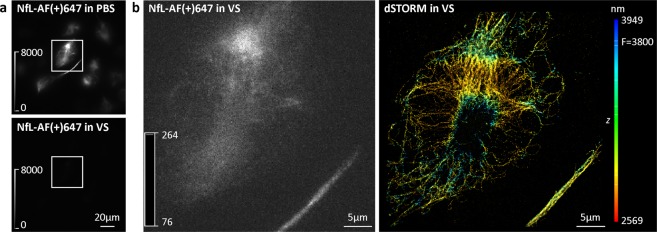
3D dSTORM super-resolution imaging of neurofilament light chain (NfL) in Vectashield (VS). (**a**) Widefield images of ND7/23 cells immunolabelled with anti-NfL primary antibody, followed by AF(+)647-conjugated secondary antibody. The same field of view is shown in PBS and in VS. To compare the effect of two different imaging media, look-up table (LUT) intensity scale bars were used. Brightness and contrast were linearly adjusted to show the same display range in both PBS and VS conditions. (**b**) TIRF image of the boxed regions in panel (**a**). An image was acquired in VS, with 647 nm laser illumination prior to dSTORM imaging. To ensure the quenched AF(+)647-labelled cell is visible, an auto-scale LUT was used. LUT intensity scale bars show minimum and maximum grey values. The corresponding 3D dSTORM image is shown on the right. The z positions in the 3D dSTORM images are colour-coded according to the height maps shown on right. Height maps contain minimal, maximal and focal (F) z position values.

**Figure 5:**
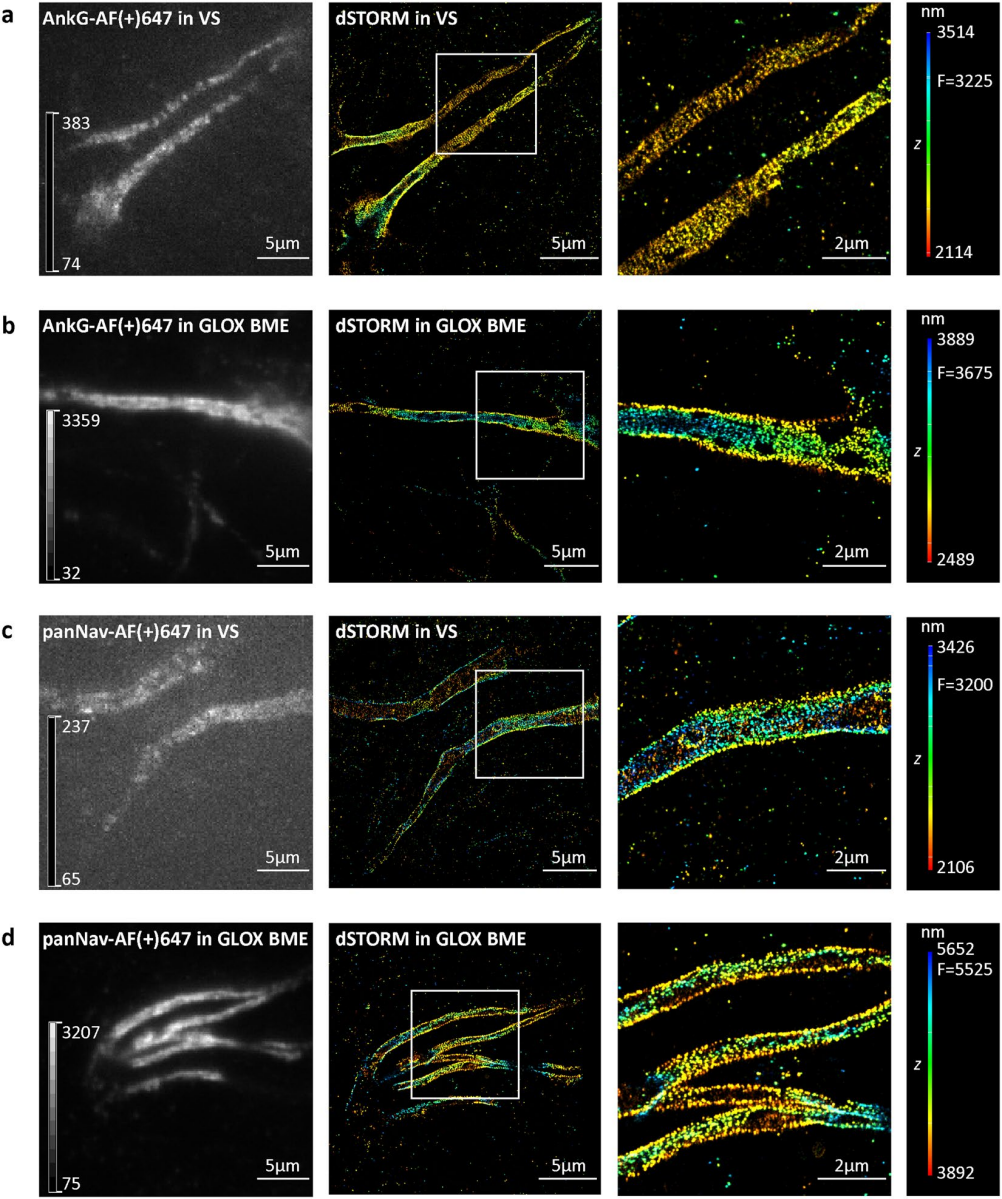
3D dSTORM super-resolution imaging of axonal initial segments in Vectashield (VS) and GLOX BME. (**a,b**) Mouse cortical neurons (MCN) labelled with anti-ankyrin G (AnkG) antibody. (**c,d**) MCN labelled with anti-pan voltage-gated sodium channel (panNav) antibody. Left panels show TIRF images, acquired with 647 nm laser illumination prior to 3D dSTORM imaging. To ensure the quenched AF(+)647 in axonal initial segments in VS is visible, an autoscale look-up table (LUT) was used. LUT intensity scale bars show minimum and maximum grey values. Middle panels show corresponding 3D dSTORM images of AnkG (**a**,**b**) and panNav (**c**,**d**). Right panels show magnification of the boxed regions shown in the middle panels. Despite the quenching effect of VS, periodic patterns of AnkG and voltage-gated sodium channels can still be resolved. The z positions in the 3D dSTORM images are colour-coded according to the height maps shown on right. Height maps contain minimal, maximal and focal (F) z position values.

To understand why the Vectashield-induced quenching has not been reported earlier, we looked at the structures that were previously successfully imaged with dSTORM in Vectashield, such as tubulin and actin^13,14,16–18^. Conventional widefield microscopy shows that AF647 is also quenched in the cells labelled with an AF647-conjugated anti-tubulin β3 antibody (Fig. 3) or AF647-phalloidin (Supplementary Fig. 6). However, AF647 quenching was less pronounced when observing these structures compared to NfL or axonal initial segments. Despite the quenching, positively labelled microtubules or actin can easily be identified with widefield microscopy. This is not the case with other proteins, such as NfL in ND7/23 cells or components of axonal initial segments, such as voltage-gated sodium channels (Nav) or ankyrin G, in primary neurons. To identify positively labelled NfL or axonal initial segments, laser illumination was required. Even using the laser and after we adjusted the image display (brightness/contrast) to show dim pixels (auto-scale look-up table), the quality of NfL (Fig. 4), ankyrin G (Fig. 5a) and Nav (Fig. 5c) labelling seems to be poor, especially when compared to the images in GLOX β-mercaptoethanol (GLOX BME) buffer (Fig. 5b,d). In addition, soon after switching to the dSTORM mode, and even under low laser illumination, we noticed that NfL-, Nav- and ankyrin G-labelled samples started blinking. This makes it challenging to search for the labelled cells and regions of interest and to set the correct focus for 3D dSTORM. However, since blinking is a prerequisite for dSTORM imaging, this prompted us to try to perform it.

For dSTORM imaging, we first identified AF(+)647-labelled NfL in PBS with conventional fluorescence microscopy. In the next step, we changed the medium to Vectashield and used laser illumination. 3D dSTORM was performed in Vectashield and we obtained SRM images with a resolution of less than 40 nm (Fig. 4), as estimated by Fourier ring correlation (FRC)^19^. However, this was only possible by first identifying the labelled cell in PBS before switching to Vectashield, that is, before the image turned dark. The same procedure also worked for the components of axonal initial segment labelled with AF(+)647, such as ankyrin G and Nav (Fig. 5).

**Figure 6:**
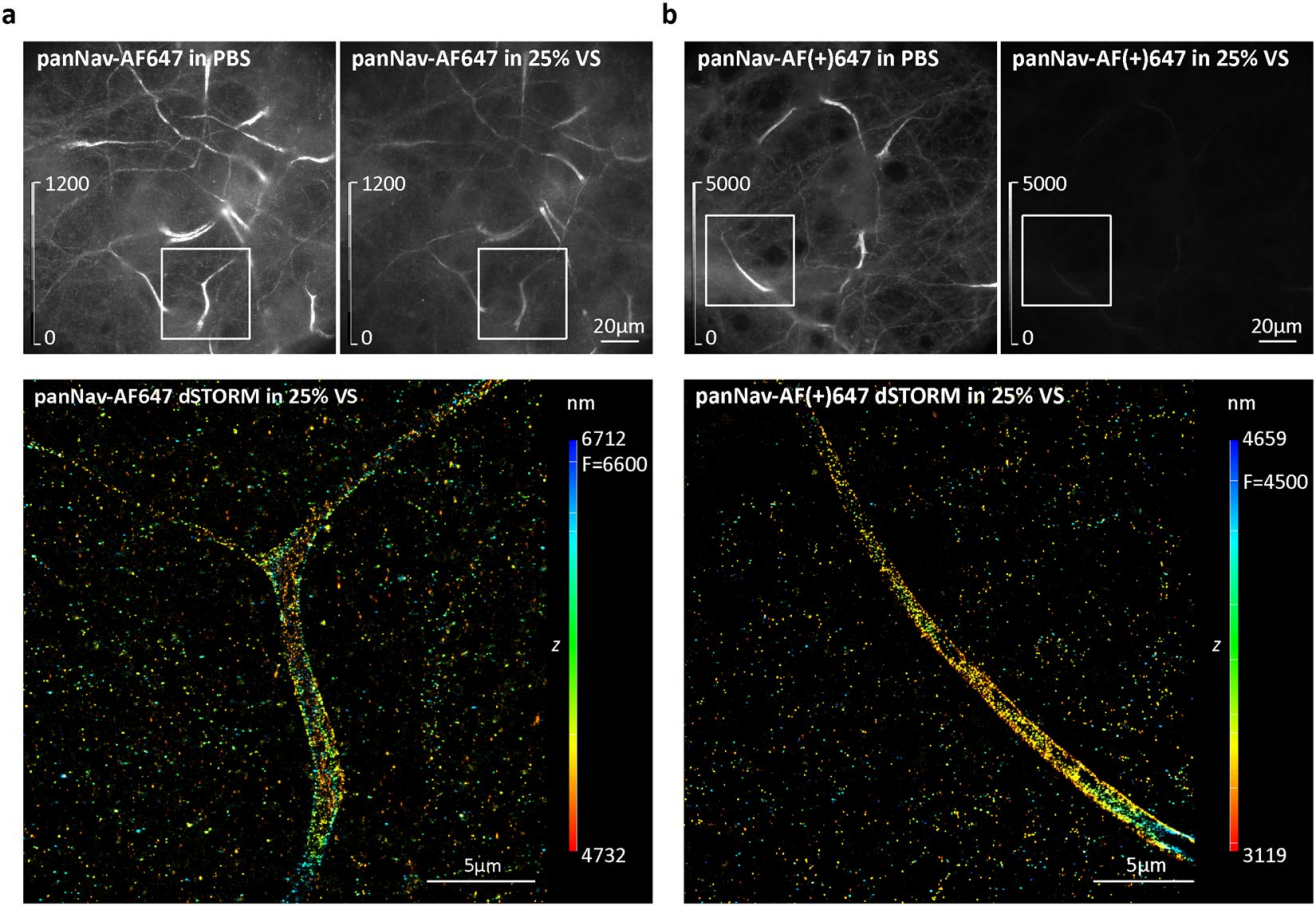
Comparison of the effect of 25% Vectashield (25% VS) on AF647 and AF(+)647 fluorescence. (**a**) Mouse cortical neurons (MCN) immunolabelled with anti-pan voltage-gated sodium channels (panNav) antibody, followed by AF647-conjugated secondary antibody. (**b**) MCN immunolabelled with panNav, followed by AF(+)647-conjugated secondary antibody. Upper panels show widefield images of the same field of view acquired in PBS and in 25% VS. Brightness and contrast were linearly adjusted to show the same display range in both PBS and 25% VS conditions (as indicated by look-up table (LUT) intensity scale bars). Lower panels show corresponding 3D dSTORM images of the boxed regions from panels (**a** and **b**). The z positions in the 3D dSTORM images are colour-coded according to the height maps shown on right. Height maps contain minimal, maximal and focal (F) z position values.

In addition to identifying the labelled cells in PBS first, and then switching to Vectashield for dSTORM, imaging could be done in 25% Vectashield (diluted in Tris-glycerol). Our quantitative analysis shows that 25% Vectashield does not induce quenching of AF647, similar to GLOX (Fig. 2d,e). This allowed us to identify labelled cells, evaluate the labelling quality and to pick brightly labelled cells, which is a prerequisite for successful dSTORM. However, this was only possible with AF647-labelled samples. As our quantitative analysis shows (Fig. 2e), AF(+)647 is quenched in both 25% Vectashield and Vectashield (Fig. 6, Supplementary Fig. 7). Despite the quenching, dSTORM imaging of AF(+)647 in 25% Vectashield was still possible, but the positively labelled cells had to be first identified in PBS (Fig. 6).

**Figure 7:**
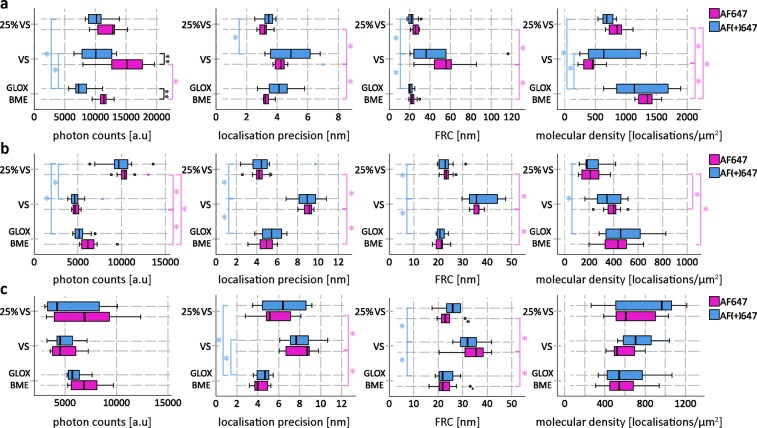
Quantification of dSTORM parameters of AF647 (magenta) and AF(+)647 (blue) in different imaging media for (**a**) tubulin β3, (**b**) βII spectrin and (**c**) neurofilament light chain (NfL). The box plots show photon counts, lateral localisation precision, FRC and molecular density in GLOX BME, 25% Vectashield (25% VS) and Vectashield (VS). For each of the parameters, 10 images per condition were analysed. Circles indicate outliers. * indicates significance with *p* < 0.05, obtained by using separate analyses of variance (ANOVAs) with post-hoc Bonferroni comparisons. ** indicates significance with *p* < 0.004167, obtained using 12 *t-*tests with Bonferroni corrections. Detailed explanations on statistical analysis can be found in the Supplementary Information.

To quantify the suitability of Vectashield for dSTORM imaging, we performed dSTORM imaging of ND7/23 cells with labelled tubulin β3 and NfL, and primary neurons with labelled βII spectrin (Supplementary Figs. 8, 9 and 10). Tubulin β3 was chosen as an example of”easy to image” high-abundance target, while βII spectrin and NfL represent less abundant targets. By less abundant we mean either biologically less abundant proteins (e.g. NfL is not so highly expressed in ND7/23 cells) or dimmer immunostainings (e.g. ßII spectrin immunostaining is dimmer than tubulin ß3). We analysed photon counts, average localisation precision, molecular density and image resolution by FRC for AF647 and AF(+)647 in different imaging media (Fig. 7). The general finding is that for all three targets Vectashield performed the worst, while 25% Vectashield gave results comparable to conventional GLOX BME buffer, but we noticed some differences in dSTORM parameters when comparing different targets, as will be discussed in detail below. Our results also show that AF647 and AF(+)647-conjugated antibodies do not differ significantly in their suitability for dSTORM.

## Discussion

### Vectashield-induced quenching of AF647 and AF(+)647 fluorescence intensity

We show that one of the most commonly used mounting media for fluorescence microscopy, Vectashield, if used as an imaging buffer, induces quenching of AF647 and its new variant, AF(+)647. As a consequence, the fluorescence emission of both AF647 and AF(+)647 is much less intense in Vectashield (about 15% and 5%, respectively, of the initial values in PBS). In contrast, AF488 fluorescence intensity is increased in both neat and 25% Vectashield (to 125% and 185%, respectively, of the initial values in PBS). As our results show, diluting Vectashield in Tris-glycerol (25% Vectashield) overcomes the problem of quenching of AF647, but not of AF(+)647.

Our findings have important consequences for any type of fluorescence microscopy. AF647 is one of the most commonly used red dyes in SRM. In combination with Vectashield, it has been used for both dSTORM and SIM^13,14,16–18,20^. However, our results suggest that AF647 and AF(+)647 in Vectashield are not optimal combinations for any type of fluorescence microscopy, because fluorescence emission of the dyes is quenched. This is especially relevant for immunohistochemical staining because Vectashield is frequently used to mount tissue sections for confocal microscopy. If it is necessary to use AF647, 25% Vectashield is a good alternative. For AF(+)647, a different mounting medium needs to be used. Alternatively, by testing further dilutions of Vectashield, it might be possible to find an optimal concentration that does not induce AF(+)647 quenching.

Even though we only quantified the effects of Vectashield and its dilution on the fluorescence emission of AF647, AF(+)647 and AF488, it is reasonable to assume that similar changes might occur with other fluorophores, antibodies, other mounting media or other dSTORM imaging buffers, especially while the composition of Vectashield remains confidential. For this reason, for any type of labelling, we would recommend first checking the cells in buffers such as PBS. This is the only way to distinguish between failed immunocytochemistry/immunohistochemistry staining and fluorescence quenching phenomena. As we show in this manuscript, even secondary antibodies of similar molecular structure (according to the manufacturer, AF647 and AF(+)647-conjugated secondary antibodies differ in their conjugating chemistry, not the actual fluorophore moiety itself) can behave differently in the same imaging medium (25% Vectashield). While Olivier and colleagues state that some dyes are not stable in Vectashield (e.g. Cy2), they do not discuss any potential stability problems with AF647 in Vectashield^13^. This was only mentioned by Cordes and colleagues who reported an 80% drop of AF647 intensity in Vectashield, which is in line with our results^15^. It was also suggested that Vectashield can induce cleavage of cyanine dyes and their derivatives. To test the hypothesis of Vectashield inducing cleavage of AF647 (a cyanine), we performed a recovery experiment in which we removed Vectashield by washing and after 2.5 h imaged the cells again in PBS. A quantitative analysis shows that the loss of AF647 fluorescence is reversible, as it can be partially recovered, which would not have been possible if the dye was cleaved. Because the dyes are affected in the ON state, Vectashield might be causing changes in the quantum yield, which directly affects the brightness of the dye.

### Procedure for dSTORM imaging of quenched AF647- and AF(+)647- labelled samples

Despite the quenching of fluorescence intensity in the ON state, AF647 and AF(+)647 dye moieties alternate between the ON and OFF states in Vectashield. Because of this, it is possible to perform dSTORM imaging in Vectashield. However, quenching of the fluorophores in the ON state hinders dSTORM imaging. This is especially relevant for less abundant targets or dimmer immunostainings as we showed in this study.

One advantage of dSTORM over other single-molecule- localisation techniques (e.g. FPALM with photoactivatable proteins) is that it uses conventional labelling and starts with the fluorophores in the ON state^21^. This enables brightly labelled cells and structures of interest to be identified. Because of quenching, this is not possible with Vectashield. In Vectashield, non-labelled (or poorly labelled cells) and quenched cells cannot be distinguished. Not being able to identify labelled cells is less critical for immunostainings of microtubules or actin filaments, which are abundant in cells. Such immunostainings typically give a homogenous signal and almost any cell can be imaged with dSTORM. These cells can even be identified with transmitted light without the need for a fluorescent signal. However, this is not possible with most of the other targets or with highly polarized cells such as neurons. One example of this is the axonal initial segment that we imaged in this study. This complex structure has a unique molecular organisation^22,23^ and it cannot be identified based on neuronal morphology and transmitted light microscopy. Under a light microscope, axonal initial segments can only be identified by labelling their specific components and identifying them with fluorescence microscopy. To image such structures with dSTORM, it is necessary to identify the positively labelled cells with conventional fluorescence prior to dSTORM imaging. For this, the dye molecules need to be in the ON state. As this is not possible in Vectashield, we identified AF647-labelled targets in PBS and switched to Vectashield for dSTORM imaging. As an alternative, 25% Vectashield can be used for AF647. According to Olivier and colleagues, diluting Vectashield in Tris-glycerol (25% Vectashield) helps to reduce Vectashield’s autofluorescent background^13^. Furthermore, our analysis shows that 25% Vectashield does not induce quenching, which alone makes it more suitable for imaging AF647 conjugates. However, according to our results, 25% Vectashield still quenched AF(+)647. Most of the studies utilizing Vectashield for dSTORM to date used 25% Vectashield (or other dilutions) and AF647. This might also explain why Vectashield-induced quenching was not previously reported. Our results show that even though both AF647 and AF(+)647 are quenched in Vectashield, they do not exhibit the same behaviour in 25% Vectashield. Because of this, additional care is necessary when choosing AF647-conjugated antibodies and imaging buffers.

### Quantifying the suitability of Vectashield for dSTORM imaging of AF647 and AF(+)647

Based on our imaging protocol, both AF647 and AF(+)647 can be used for dSTORM in Vectashield. However, depending on the target, imaging in Vectashield is more or less optimal. It is important to reiterate that abundant cytoskeletal elements such as microtubules were readily imaged in Vectashield, but problems emerged when imaging other proteins, such as NfL in neuronal cells and voltage-gated sodium channels, ankyrin G and βII spectrin in primary neurons. This is probably a consequence of the relatively higher starting labelling intensity of microtubules. For example, anti-tubulin ß3 immunocytochemistry shows relatively nicely labelled cells in Vectashield. Without seeing the image in PBS (before the medium exchange for Vectashield), one might not even realize that there was quenching. Thus, depending on the target itself and if it’s a high- or low-abundance protein, the quality of the antibody and the labelling protocols (brighter or dimmer immunostaining), the quenching effect of Vectashield might be more or less apparent. Nevertheless, the quality of dSTORM of AF647-labelled samples in Vectashield is not the same as in GLOX BME. Comparison of dSTORM parameters shows that Vectashield performs worse than GLOX BME, whereas 25% Vectashield gave results comparable to GLOX BME. For tubulin β3, this is reflected by differences in lateral localisation precision, image resolution (estimated by FRC) and molecular density. Vectashield gave worse average lateral localisation precision, higher FRC values (lower image resolution) and lower molecular density compared to GLOX BME and 25% Vectashield. 25% Vectashield and GLOX BME performed the same except in terms of molecular density, which was slightly higher for GLOX BME than for 25% Vectashield. We also looked at the photon counts. In the case that the number of photon counts correlates with image quality, using Vectashield could be expected to yield the lowest photon counts. Instead, Vectashield gave the highest photon counts. However, because the identified molecules have lower density and worse localisation precision compared to GLOX BME and 25% Vectashield, they afforded lower image resolution. One of the problems with imaging of AF647 in Vectashield was the inability to get all the molecules to blink efficiently, which also explains the high variance in Vectashield FRC values. In some cases, we achieved good resolution (low FRC values) and in others not.

For AF647-labelled βII spectrin, we see more or less the same trend as for tubulin β3. In general, neat Vectashield performed worse than 25% Vectashield and GLOX BME. FRC values were higher and localisation precision was worse in Vectashield. In contrast to tubulin β3, we observed a different trend in molecular density- molecular density in 25% Vectashield was lower than in Vectashield or GLOX BME. Photon counts also show a different trend. For AF647-labeled βII spectrin, we get the highest photon counts in 25% Vectashield, followed by GLOX BME and then Vectashield.

For AF647-labelled NfL, we see more or less the same trend as for tubulin β3 and βII spectrin. In general, neat Vectashield performed worse than 25% Vectashield and GLOX BME. FRC values were higher and localisation precision was worse in Vectashield. In contrast to tubulin β3 and βII spectrin, we observed no significant changes in molecular density and photon counts. Instead, these values had a very large variation, which is probably a consequence of NfL-labelled samples being most affected by the Vectashield-induced quenching.

We also compared the two dyes and the general conclusion is that in each of the imaging media for all three targets (tubulin β3, βII spectrin, NfL) they behave more or less the same. The only significant difference was for AF647-labeled tubulin β3 photon counts: AF647 gives a higher number of photon counts than AF(+)647 in Vectashield and GLOX BME. As discussed above, we do not fully understand the implications of these findings. Under our imaging conditions and with our analysis, higher photon counts do not correlate with better image resolution of AF647-labeled tubulin β3. Higher photon counts actually made the analysis of tubulin β3-labelled cells more difficult- we could not use the same thresholding value for molecule identification in different imaging media. Instead we had to use automatic minimum heights for molecule identification. We also tried to use fixed thresholding values for molecule identification. This resulted in identifying more molecules but with worse localisation precision, so that the end result was the same- the worst image quality (highest FRC values) was with Vectashield (data not shown). As also mentioned above, we see different trends in photon counts changes in different imaging media for different targets, but this does not seem to correlate with the changes in image resolution (as estimated by FRC). Instead, changes in FRC seem to correspond to the changes in localisation precision and to some extent molecular density.

Until now, mainly cytoskeletal elements, such as tubulin and actin, have been imaged in Vectashield^13,14,16–18^. Most biological structures are not as abundant as microtubules, so we predict that other targets might be affected similarly to those we imaged in this study. With our imaging protocol and quantitative analysis, it should be possible to optimize the imaging conditions of any target in Vectashield. Furthermore, as our analysis shows, 25% Vectashield performed very well compared to GLOX BME. This was true for both AF647 and AF(+)647. Interestingly, our results also suggest that quenching does not necessarily lead to lower quality dSTORM images. For example, AF(+)647 is quenched in 25% Vectashield but still gives good FRC values, even for the low-abundance target, such as NfL. In this case, quenching is a problem for identifying cells/regions of interest and is hindering the process of dSTORM imaging, but does not seem to influence dSTORM parameters; at least, this is true for our imaging approach. If one were to try to “blindly” image quenched cells, imaging parameters might not be the same.

## Methods

### Cell culture

Mouse neuroblastoma x rat neuron hybrid ND7/23 cells (ECACC 92090903, Sigma Aldrich) were grown in high glucose Dulbecco’s Modified Eagle’s Medium (DMEM; Thermo Fisher Scientific, cat. no. 41965062) supplemented with 10% heat-inactivated fetal bovine serum (FBS; Thermo Fisher Scientific, cat. no. 10270106), 1% penicillin-streptomycin (Sigma Aldrich, cat. no. P0781), 1% sodium pyruvate (Thermo Fisher Scientific, cat. no. 11360039) and 1% L-glutamine (Thermo Fisher Scientific, cat. no. 25030024) at 37 °C, 5% CO_2_. FBS was heat-inactivated by incubation at 56 °C for 30 minutes (min). For more details, see Supplementary Information.

Primary mouse cortical neurons (MCN) from C57BL/6 embryonic day-17 mice were obtained from Thermo Fisher Scientific (cat. no. A15586). Neurons were thawed following the manufacturer’s recommendations and seeded in eight-well Lab-Tek II chambered #1.5 German coverglass (Thermo Fisher Scientific, cat. no. 155409). Neurons were maintained in B-27™ Plus Neuronal Culture System (Thermo Fisher Scientific, cat. no. A3653401), supplemented with 1% penicillin-streptomycin (Sigma Aldrich, cat. no. P0781). Half of the culturing medium was changed twice per week.

### Constructs and cloning

NLS (nuclear localisation sequence)-mCherry construct was made by inserting NLS-mCherry sequence into a commercially available pcDNA^TM^3.1/Zeo(+) mammalian expression vector (Invitrogen). NLS sequence was added upstream of the mCherry gene (gift from Edward Lemke’s laboratory, EMBL, Heidelberg) by polymerase chain reaction (PCR). The following primers were used for cloning: 5′-GCTGGCGCTAGCACCATG**CCGCCGAAAAAAAAACGCAAAGTGGAAGATAGC**GTGAGCAAGGGCGAGGAGG-3' (forward primer with NheI restriction site; NLS sequence is shown in bold) and 5′-CGCGCAGCGGCCGCTCACTTGTACAGCTCGTCCATGCCG-3' (reverse primer with NotI restriction site). Generated plasmid sequence was confirmed by sequencing.

### Transfections (for AF647, AF(+)647 and AF488 intensity measurements)

For Alexa Fluor 647 (AF647), Alexa Fluor Plus 647 (AF(+)647) and Alexa Fluor 488 (AF488) intensity measurement experiments, ND7/23 cells were transfected with NLS-mCherry plasmid. Cells were transfected using JetPrime (Polyplus-transfection, cat. no. 114 - 15) one day after seeding, at 80–85% confluence, according to the manufacturer´s instructions. On the following day, NLS-mCherry expression was confirmed on an epifluorescent inverted microscope (Olympus CKX41) and immunostaining was performed.

### Immunocytochemistry stainings

For immunocytochemistry (ICC) stainings, ND7/23 cells were washed briefly with 0.01 M phosphate-buffered saline (PBS; 137 mM NaCl, 10 mM Na2HPO4, 1.8 mM KH2PO4, 2.7 mM KCl, pH 7.4). Tris-buffered saline (TBS; 20 mM Tris, 150 mM NaCl, pH 7.6) was used instead of PBS for immunostaining of neurofilament light chain (NfL) because of potential influence of PBS on phosphorylated neurofilaments. Afterwards, cells were fixed with 2% paraformaldehyde (PFA; Sigma Aldrich, cat. no. 158127) at RT. For labelling of tubulin β3, cells were fixed as described previously^13^.

After fixation, cells were briefly washed again, blocked and permeabilized. Following primary antibodies were used: anti-tRFP (turbo red fluorescent protein) antibody (Evrogene, cat. no. AB233), mouse anti-neurofilament 70 kDa antibody, clone DA2 (Merck Millipore, cat. no. MAB1615), AF647-conjugated anti-Tubulin β3 (TUBB3) antibody (BioLegend, cat. no. 801210), AF488-conjugated anti-TUBB3 antibody (BioLegend, cat. no. 801203), anti-TUBB3 antibody (BioLegend, cat. no. 801202). For actin labelling, cells were incubated with phalloidin AF647 (Thermo Fisher Scientific, cat. no. A22287).

The following secondary antibodies were used: goat-anti rabbit AF647 (Thermo Fisher Scientific, cat. no. A-21245), goat anti-rabbit AF647 Plus (Thermo Fisher Scientific, cat. no. A32733), goat anti-rabbit AF488 (Thermo Fisher Scientific, cat. no. A-11034), goat anti-mouse AF647 Plus (Thermo Fisher Scientific, cat. no. A32728), goat anti-mouse AF488 Plus (Thermo Fisher Scientific, cat. no. A32723), goat anti-mouse AF647 (Thermo Fisher Scientific, cat. no. A21236), goat anti-mouse AF555 (Thermo Fisher Scientific, cat. no. A21424), goat anti-mouse AF633 (Thermo Fisher Scientific, cat. no. A21052).

MCN were fixed on various days *in vitro* (DIV) with 4% EM grade PFA (Electron Microscopy Sciences, cat. no. 15710) diluted in PEM buffer (80 mM PIPES, 2 mM MgCl_2_, 5 mM EGTA, pH 6.8) for 15 minutes at RT. After fixation, background fluorescence was quenched with sodium borohydride (Sigma Aldrich, cat. no. 71320), cells were washed 3 times (10 minutes each wash) with PBS, blocked and permeabilized. The following primary antibodies were used: mouse monoclonal anti-pan sodium channel antibody (panNav; Sigma Aldrich, cat. no. S8809), mouse monoclonal anti-ankyrin G antibody (Santa Cruz, cat. no. sc-12719) and mouse monoclonal anti-beta-spectrin II (βII spectrin) clone 42 (BD Biosciences, cat. no. 612 563). Goat anti-mouse secondary antibodies conjugated with AF647 Plus or AF647 were used.

Details on ICC staining steps and antibodies used in each figure are provided in Supplementary tables S1, S2 and S3.

After labelling, ND7/23 cells and MCN were washed 3 times (5 minutes each wash) and imaged on the same day. Cells stained with anti-tRFP (NLS-mCherry transfected cells) were also imaged on the following day.

### Microscope configuration

Widefield epifluorescence and 3D dSTORM imaging were performed on an N-STORM 4.0 microscope from Nikon Instruments. More specifically, this is an inverted Nikon Eclipse Ti2-E microscope (Nikon Instruments), equipped with XY-motorized stage, Perfect Focus System, an oil-immersion objective (HP Apo TIRF 100×H, NA 1.49, Oil) and N-STORM module. Setup was controlled by NIS-Elements AR software (Nikon Instruments). Fluorescent light was filtered through the following filter cubes: 488 (AHF; EX 482/18; DM R488; BA 525/45), 561 (AHF; EX 561/14; DM R561; BA 609/54), Cy5 (AHF; EX 628/40; DM660; BA 692/40) and Nikon Normal STORM cube (T660lpxr, ET705/72 m). Filtered emitted light was imaged with ORCA-Flash 4.0 sCMOS camera (Hamamatsu Photonics). For epifluorescent widefield imaging, fluorescent lamp (Lumencor Sola SE II) was used as a light source. For 3D dSTORM imaging, a 647 nm laser (LU-NV Series Laser Unit) was used and a cylindrical lens was introduced in the light path^11^.

### Imaging of tRFP labelled cells (for AF647, AF(+)647 and AF488 intensity measurements)

tRFP labelled cells were first briefly checked in PBS using brightfield illumination. For each well in the Lab-Tek we did the following: picked randomly 30 fields of view (stage positions) using brightfield illumination, saved xyz coordinates of each field of view in NIS-Elements AR software and acquired images automatically by using NIS-Elements ND multipoint acquisition module, which allowed us to go to the same position each time. The images were acquired in widefield mode, with 10 ms exposure time, 1024 × 1024 pixels frame size and 16-bit image depth. To provide that the cells were always properly focused, we used an autofocusing function of ND multipoint acquisition module.

Imaging was done first in PBS, using 561 (mCherry channel) and either 488 (AF488 channel) or Cy5 (AF647 channel) filter cube, depending on the labelling condition. Excitation light intensity for mCherry and AF647 channels was 10% and for AF488 channel 5%. Afterwards, PBS was replaced with one of the following imaging media: PBS, 100% Vectashield (Biozol, cat. no. VEC-H-1000), 25% Vectashield or GLOX. More details on imaging media composition can be found in Supplementary Information. For AF647 recovery experiments, 100% Vectashield was removed and cells were washed twice with PBS. After 2.5 h, cells were washed one more time with PBS and imaging was repeated. To provide enough data for analysis, each experiment was repeated at least three times for AF488, AF647 and AF(+)647 labelled cells.

### Image analysis and intensity measurements

Intensity measurements for quantitative analysis of AF647, AF(+)647 and AF488 were done in Fiji/ImageJ^24^. Images in ND2 format were opened using Bio-Formats plugin^25^ and converted to tiff before the analysis. Original bit depth (16-bit) was used for analysis. For presentation purposes, brightness and contrast of 16-bit images were linearly adjusted in Fiji or NIS Elements, look-up table (LUT) intensity bars were added in NIS Elements, images were converted to 8-bit tiff format and imported in Adobe Illustrator.

More details on image analysis and intensity measurements are provided in Supplementary Information.

### 3D dSTORM imaging

3D dSTORM (direct stochastic optical reconstruction microscopy) imaging was performed by using the N-STORM module of Nikon microscope described above. For imaging, oil-immersion objective (HP Apo TIRF 100 × H, NA 1.49, Oil) and 647 nm laser (LU-NV Series Laser Unit) were used. Fluorescent light was filtered through a Nikon Normal STORM filter cube. Filtered emitted light was imaged with ORCA-Flash 4.0 sCMOS camera (Hamamatsu Photonics) with a cylindrical lens introduced in the light path^11^.

Fluorescent and brightfield images of labelled cells in PBS and Vectashield were acquired using fluorescent light source and Cy5 filter cube or 488 filter cube (more details are provided in Supplementary Information). After widefield acquisition, NfL and tubulin β3 labelled cells were imaged with 3D dSTORM. Imaging was performed in total internal reflection fluorescence (TIRF) or highly inclined and laminated sheet microscopy (HiLo) mode with continuous 647 nm laser illumination (full power). Frame size was 256 × 256 pixels and image depth 16-bit. For each 3D dSTORM image, 30,000 frames were acquired at 33 Hz.

Imaging of axon initial segments was performed in the way described for NfL and tubulin β3, with some modifications. For imaging of sodium channels, 30,000 frames were acquired at 50 Hz or 33 Hz, and for imaging of ankyrin G, 20,000 or 30,000 frames were acquired at 25 Hz or 33 Hz. 3D STORM imaging was done in 100% Vectashield, 25% Vectashield or GLOX β-mercaptoethanol (GLOX BME).

Calibration of 3D dSTORM was done previously using TetraSpeck Microspheres (Thermo Fisher Scientific, cat. no. T7279) following NIS-Elements’ instructions. 3D dSTORM image processing was performed in NIS-Elements AR software. Molecule identification settings were set to defaults for 3D dSTORM analysis: minimum width 200 nm, maximum width 700 nm, initial fit width 300 nm, max axial ratio 2.5 and max displacement 1. Minimum height for peak detection was set to 200 (NfL), 750 (tubulin β3), 100 (Nav) and 500 (ankyrin G), and localisation analysis was performed with overlapping peaks algorithm and drift correction. Resulting molecule lists were exported as text files and analysed by Fourier ring correlation (FRC)^19^ to determine the image resolution. 3D dSTORM images of tubulin β3, NfL, ankyrin G and sodium channels were reconstructed with Gaussian rendering size of 15 nm in NIS Elements AR and final 3D dSTORM images (with z position height maps) were exported as tiff files. Z rejected molecules were excluded from resolution analysis and from final images.

### dSTORM imaging for quantitative analysis of dSTORM quality

For quantitative analysis of dSTORM quality, imaging was performed in ND7/23 cells stained with tubulin β3 and NfL antibodies and mouse cortical neurons stained with βII spectrin antibody in the way it was described above, with some modifications. Frame size was 128 × 128 pixels for all targets, tubulin β3, NfL and βII spectrin. For 3D dSTORM imaging of tubulin β3, 20,000 frames were acquired at 33 Hz, and for 3D dSTORM imaging of NfL and βII spectrin, 30,000 frames were acquired at 33 Hz. Both AF647 and AF(+)647 labelled cells were imaged on the same day, in 100% Vectashield, 25% Vectashield and GLOX BME. To provide enough data for analysis, each experiment was repeated three times. At least 10 3D dSTORM images were acquired in each imaging medium for both dyes (AF647 and AF(+)647), and for all targets, tubulin β3, NfL and βII spectrin. Raw image data used for dSTORM quantification are available in the BioImage Archive^26,27^ (http://www.ebi.ac.uk/bioimage-archive) under accession number S-BIAD16.

### Quantitative analysis of dSTORM quality

For quantitative analysis of dSTORM quality we determined the following properties of AF647 and AF(+)647 dyes: total number of photons received from molecule across its entire trace length (photon counts), total number of molecules, lateral localisation accuracy, the number of localisations per square micrometer (µm^2^; molecular density), and the resolution (FRC) of 3D dSTORM images.

For quantitative 3D dSTORM analysis, image processing was done in NIS-Elements AR software. Molecule identification settings were set to defaults for the analysis: minimum width 200 nm, maximum width 700 nm, initial fit width 300 nm, max axial ratio 2.5, max displacement 1, and maximum height 65000. Minimum height for peak detection was set to 400 (NfL and βII spectrin, data not shown), 500 (tubulin β3, data not shown) or automatic minimum height (tubulin β3, NfL and βII spectrin, data shown in the manuscript), and localisation analysis was performed with the overlapping peaks algorithm and drift correction. Due to incomplete blinking, the first 150 frames (NfL and βII spectrin) or 300 frames (tubulin β3) were excluded from the analysis. Resulting molecule lists were also filtered to exclude Z rejected molecules and exported as text files. Text files were imported in Igor Pro and the average values of photon counts and lateral localisation accuracy were calculated for each dSTORM image. To determine dSTORM image resolution, filtered molecule lists were analysed by Fourier ring correlation (FRC). Based on the filtered molecule lists, representative 3D dSTORM images of tubulin β3, NfL and βII spectrin were reconstructed with Gaussian rendering size of 4 nm in NIS Elements AR and final images (with z position height maps) were exported as tiff files. For molecular density calculations, filtered molecule lists were displayed as Density Maps in NIS Elements AR and exported as tiff files. Resulting tiff images were imported in ImageJ and the area fraction of non-zero pixels was measured. This number was used to calculate the physical area (in µm^2^) of non-zero pixels. As a final step, we divided the total number of molecules (localisations) with the physical area. The resulting number represents the molecular density (number of molecules/µm^2^).

### Statistical analysis

Statistical analyses (t-test, ANOVA, post-hoc analyses, box plots) were carried out with IBM SPSS Statistics Version 25, Armonk, New York, USA. The power analysis to determine the optimal sample size was carried out with the Statistical Tree Power Calculator, QFAB (Queensland Facility for Advanced Bioinformatics), Brisbane, Australia. Further details on statistical analysis are in Supplementary Information.

## Supplementary information

Supplementary Information.

## Data Availability

Raw image data used for dSTORM quantification reported in Fig. 7 of this paper are available in the BioImage Archive (http://www.ebi.ac.uk/bioimage-archive) under accession number S-BIAD16. Remaining datasets are available on request from the corresponding author.
